# Modulation of Myeloid-Derived Suppressor Cells in the Tumor Microenvironment by Natural Products

**DOI:** 10.1007/s00005-023-00681-0

**Published:** 2023-07-06

**Authors:** Jhon Jairo Calderon, Karol Prieto, Paola Lasso, Susana Fiorentino, Alfonso Barreto

**Affiliations:** 1 Grupo de Inmunobiología y Biología Celular, Facultad de Ciencias, Pontificia Universidad Javeriana, Bogotá, Colombia; 2 Departamento de Microbiología, Pontificia Universidad Javeriana, Carrera 7 # 43-82. Edificio 50 Laboratorio 101, Bogotá, Colombia

**Keywords:** Myeloid-derived suppressor cells, MDSCs, Tumor microenvironment, Cancer, Natural products, Phytotherapy

## Abstract

During carcinogenesis, the microenvironment plays a fundamental role in tumor progression and resistance. This tumor microenvironment (TME) is characterized by being highly immunosuppressive in most cases, which makes it an important target for the development of new therapies. One of the most important groups of cells that orchestrate immunosuppression in TME is myeloid-derived suppressor cells (MDSCs), which have multiple mechanisms to suppress the immune response mediated by T lymphocytes and thus protect the tumor. In this review, we will discuss the importance of modulating MDSCs as a therapeutic target and how the use of natural products, due to their multiple mechanisms of action, can be a key alternative for modulating these cells and thus improve response to therapy in cancer patients.

## Introduction

Myeloid cells comprise a diverse group of immune cells, primarily including macrophages, monocytes, dendritic cells (DCs), and polymorphonuclear (PMN) leukocytes. These cells can respond to signals and stimuli from other cells, leading to their activation and acquisition of antigen-presenting and pro-inflammatory functions. In some instances, they can also be activated and exert an immunosuppressive and anti-inflammatory function (Bassler et al. 2019). Under normal circumstances, myeloid cells are derived from a common myeloid progenitor in the bone marrow (BM), which gives rise to a granulocyte–monocyte progenitor (GMP). The GMP further differentiates into a monocyte/DC progenitor or a myeloblast, ultimately maturing into monocytes/DCs or PMNs, respectively (Groth et al. 2019; Passegué et al. 2003).

**Fig. 1:**
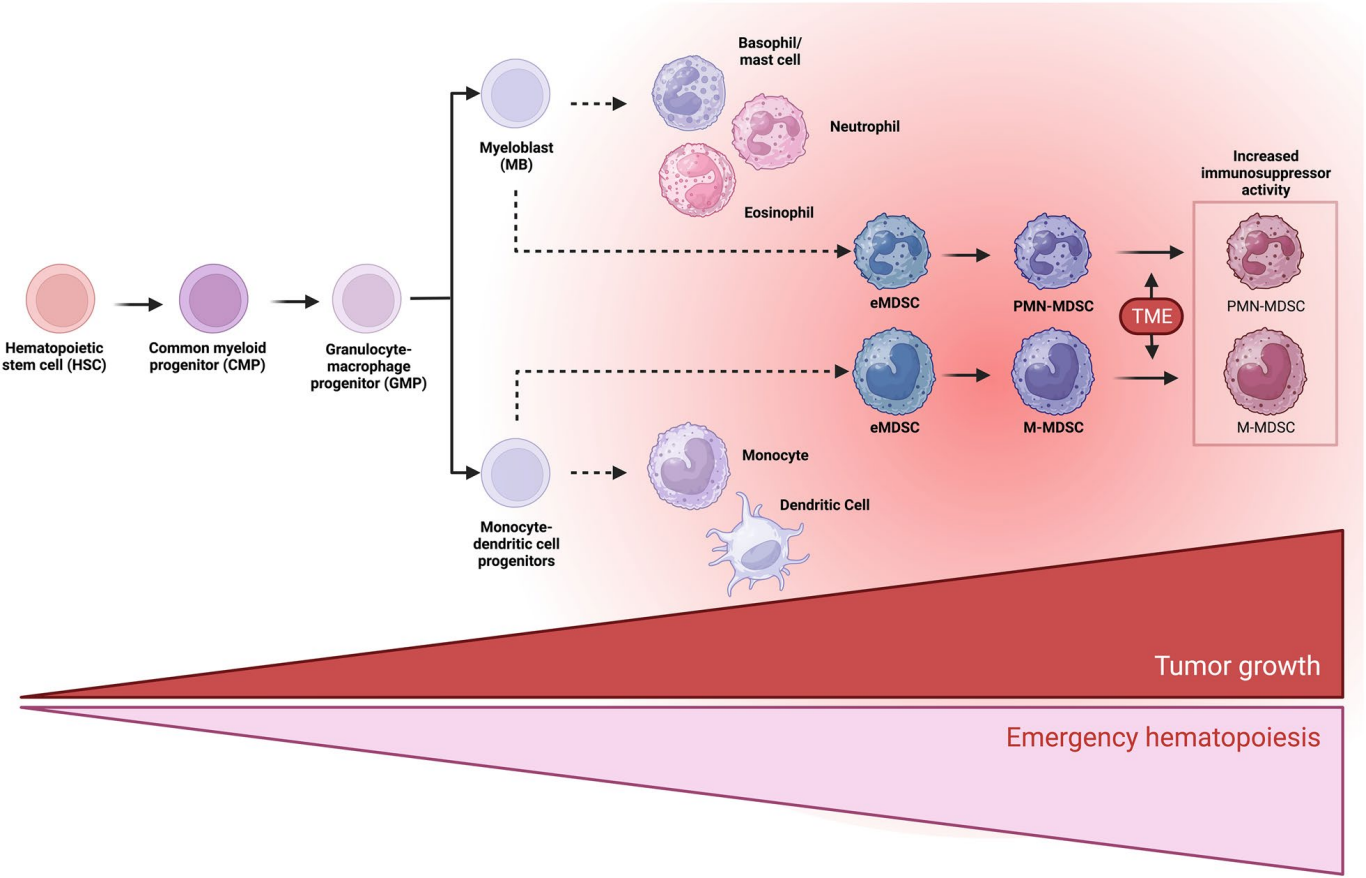
MDSC differentiation. In normal myelopoiesis, hematopoietic stem cells (HSC) differentiate into a common myeloid progenitor (CMP), which will give rise to granulocyte and monocyte progenitor cells (GMP), will differentiate into myeloblasts (MB) or macrophage/dendritic progenitor cells (MDP), and which will give rise to granulocytes, macrophages, and dendritic cells respectively. However, during chronic inflammatory processes such as in cancer, there is a constant stimulus to the bone marrow, inducing an emergency hematopoiesis that increases the migration of undifferentiated cells and results in an accumulation of heterogeneous myeloid cells with phenotype and suppressive activity, called MDSCs, which are activated and enhance their suppressive capacity by infiltrating the TME

Myelopoiesis is a tightly regulated process that can be modulated by several conditions, such as inflammation. During acute processes, such as injuries or infections, hematopoietic cells are recruited to infiltrate and solve the problem. This leads to a significant decrease in circulating cells. Therefore, the BM initiates an “emergency myelopoiesis” response (Groth et al. 2019; Pietras 2017). Conversely, chronic inflammatory conditions such as cancer result in a drastic and constant decrease in peripheral myeloid cells. This, coupled with elevated cytokine levels and tumor-derived factors, contributes to the generation of immature myeloid cells with an immunosuppressive phenotype. For this reason, they are called myeloid-derived suppressor cells (MDSCs) (Groth et al. 2019; Loftus et al. 2018; Millrud et al. 2017; Sendo et al. 2018) (Fig. 1).

MDSCs are naturally suppressive as they exhibit features such as arginase 1 (Arg1) expression, inducible nitric oxide synthase (iNOS), and molecules such as programmed death-ligand 1 (PD-L1), among other immunosuppressive molecules. However, when they infiltrate the tumor microenvironment (TME), these characteristics are exacerbated due to the adverse conditions that occur in tumor, such as oxidative stress and inflammation, which leads to the activation of cellular stress pathways such as endoplasmic reticulum (ER) stress, which has been postulated as a fundamental pillar to favor the polarization of MDSCs (Hetz and Papa 2018; Lee et al. 2014). To achieve a better response to TME, MDSCs have been proposed as a crucial therapeutic approach. Several strategies have been suggested to modulate their activity, that in the case of natural products, those abundant in polyphenols have been tested. These compounds, such as Green Tea (Xu et al. 2020), have demonstrated the ability to affect their suppressive functions and infiltration within the TME, as well as a plant extract with a major number of compounds from *Caesalpinia spinosa* (Lasso et al. 2022). Harnessing the potential of these natural products holds considerable promise as novel therapies aimed at combating these specific cell types.

## Characteristics and Signaling Pathways Involved in the Generation of MDSCs

MDSCs in mice were characterized first by their high expression of Mac-1 (CD11b) and Gr-1 (the anti-Gr1 antibody recognizes both Ly6C and Ly6G antigens). However, recent studies have revealed the presence of distinct subpopulations within these MDSCs, which could be defined as polymorphonuclear MDSCs (PMN-MDSCs) or monocytic MDSCs (M-MDSCs) (Table 1). Human MDSCs have equivalent populations with the addition of one population denominated early-stage MDSC (eMDSCs) with different phenotypical characteristics that represent myeloid precursors (Table 1) (Bronte et al. 2016; Veglia et al. 2021). In the ongoing research on MDSCs, several novel markers like CD84, IL-1ß, CD66b, S100A9, and others are emerging to distinguish between PMN-MDSCs from M-MDSCs and neutrophils (Li et al. 2021).

**Table 1:** Phenotypic characteristics to identify MDSCs

	Mouse	Human
PMN-MDSCs	CD11b^+^Ly6G^+^Ly6C^low^	CD11b^+^CD14^−^ CD15^+^/CD66b^+^ LOX1^+^
M-MDSCs	CD11b^+^Ly6G^−^Ly6C^hi^	CD11b^+^CD14^+^HLA-DR^−/low^ CD15^−^
eMDSCs	–	Lin^−^ (CD3/14/15/19/56)/HLA-DR^−^/CD33^+^

*eMDSCs* early-stage MDSC, *M-MDSCs* monocytic MDSCs

The role of MDSCs in cancer progression involves two initial steps: mobilization and migration to the TME. The mobilization process relies on a variety of inflammatory mediators produced by tumor cells, including granulocyte–macrophage colony-stimulating factor, granulocyte colony-stimulating factor (G-CSF), macrophage colony-stimulating factor, stem cell factor, vascular endothelial growth factor (VEGF), transforming growth factor (TGF)-β, tumor necrosis factor (TNF)-α, interleukin (IL)-1β, IL-6, IL-10, and other transcription factors released that induce rapid myelopoiesis in both BM but also lymphoid nodules. They also contribute to the blockade of myeloid cell maturation and the initiation of immunosuppressive pathways (Gabrilovich et al. 2012; Hao et al. 2021; Karin 2020; Veglia et al. 2021). Recruitment is influenced by multiple pro-inflammatory chemokines, including CXCL1/CXCL2/CXCL3 with receptor CXCR 2, CXCL12 with CXCR 4, and CXCL8 (IL-8) with receptor CXCR1/CXCR (Highfill et al. 2014; Li et al. 2019, 2020). Therefore, these molecules involved both in mobilization from the BM and in migration to tissues, such as the spleen, lymph nodes, or tumors, constitute attractive targets for antitumor therapies.

Expansion and activation of MDSCs in the TME are also dependent on cytokines secreted by tumor cells, such as interferon (IFN)-γ, IL-1β, IL-4, and IL-6 or activated immune cells like GM-CS, G-CSF, and VEGF (Condamine and Gabrilovich 2011). Subsequent molecular events are associated with STAT signaling pathways, where STAT3 activation is one of the most significant steps (Condamine and Gabrilovich 2011; Poschke et al. 2010). S100A9 and S100A8, known to inhibit DC maturation and increase MDSCs accumulation, have been associated with STAT3 activation in hematopoietic progenitors (Cheng et al. 2008; Condamine and Gabrilovich 2011). Activation of other proteins in the STAT family has also been linked to MDSCs suppressive activity. STAT1 signaling increases iNOS, Arg1, and PD-L1 (Gallina et al. 2006; Veglia et al. 2021). Further, inhibition of STAT5 with sunitinib reduces MDSCs accumulation and restores T cell activity in mice models (Condamine and Gabrilovich 2011; Ko et al. 2010). Additionally, STAT6 knockout mice exhibited diminished Arg1 expression and decreased suppressive activity in MDSCs (Aboelella et al. 2021; Munera et al. 2010). This highlights the importance of signaling factors, such as STAT1, STAT3, STAT5, and STAT6, in MDSCs accumulation and function. Furthermore, other pathways, including the tumor-derived exosome-associated protein HSP70 inducing MDSCs suppressive activity through TLR2/MyD88 signaling and autocrine IL-6 production (Condamine and Gabrilovich 2011; Martino et al. 2010; Millrud et al. 2017), as well as the activation of NF-κB pathway and other factors, such as IRF8 and Notch, have been implicated in the generation and functionality of these cells (Condamine et al. 2015; Netherby et al. 2017).

## Immunosuppression Mechanisms Associated with MDSCs

MDSCs employ a variety of immunosuppressive mechanisms, in the context of anti-tumor immunity, which can be categorized into four main types: production of reactive oxygen species/reactive nitrogen species (ROS/RNS), generation of cytokines and immunosuppressive mediators, metabolite depletion, and expression of immune checkpoint molecules.

### ROS/RNS Generation

MDSCs can induce a highly oxidative microenvironment primarily through increased iNOS and NADPH oxidase (NOX2) expression. iNOS is the one of the principal mechanisms by which MDSCs generate nitric oxide (NO) by decomposition of L-arginine, which plays a fundamental role in disturbing antigen presentation by DC through nitration of Tyr-701 of STAT1, preventing its phosphorylation and inhibiting antigen presentation (Markowitz et al. 2017). Additionally, NO can react with the superoxide anion generating peroxynitrite, which favors the nitration of the tyrosines present in the T cell receptor (TCR), causing a significant rigidity of these molecules and therefore hindering its interaction with MHC molecules affecting the activation of T lymphocytes and T cell apoptosis (Groth et al. 2019; Nagaraj et al. 2007; Veglia et al. 2021; Yang et al. 2020). On the other hand, ROS produced by MDSCs can give rise to hydrogen peroxide, hydroxyl radical, and hypochlorous acid, which can generate damage at the level of proteins, DNA, and lipids, as well as decrease the ζ chain of the TCR and production of IFN-γ, leading to apoptosis of T lymphocytes and, as a consequence, to a deficient antitumor activity (Corzo et al. 2009; Groth et al. 2019; Ohl and Tenbrock 2018). To prevent this phenomenon, some works described that some natural products, particularly containing high levels of polyphenols such as olive leaf extract, can decrease ROS production in MDSCs by decreasing iNOS mRNA levels, resulting in a lower RNS production in these cells (Ashourpour et al. 2016). In general terms, the attenuation of ROS levels on cancer cells or TME MDSCs indirectly or directly can impact tumor growth by decreasing the level of intra-tumor activation of MDSCs. Due to this fact, several of the potential treatments against MDSC fall on molecules with antioxidant potential, although they really do not constitute a mechanism directed specifically toward MDSCs.

### Production of Cytokines and Immunosuppressive Mediators

MDSCs produce a wide range of cytokines with different types of functions that have been described, including IL-10 and TGF-β, which are involved in the polarization of immune cells to immuno-suppressor phenotype, favoring the expression of FoxP3 to generate regulatory T cells (Groth et al. 2019; Huang et al. 2006) or diverting macrophages from an M1 to an M2 phenotype (Beury et al. 2014; Groth et al. 2019; Srivastava et al. 2012). Additionally, in a mouse model of oral squamous cell carcinoma, TGF-β is highlighted as a fundamental factor to enhance the infiltration and functionality of M2 macrophages (Maldonado et al. 2022). TGF-β is also involved in the immune suppression of NK cell cytotoxicity (Fleming et al. 2018), where the importance of SMAD3 signaling in this suppression process has been demonstrated (Chung et al. 2021; Tang et al. 2017) while TGF-β and IL-10 support Breg cells proliferation as well (Shen et al. 2018). It is important to bring up that the treatment with polyphenols such as resveratrol, in a lung cancer model, demonstrated a decrease in STAT3, preventing the functionality of the Breg cells (Lee-Chang et al. 2013). Additionally, MDSCs are very important in the production of pro-inflammatory cytokines, such as IL-1β and IL-6, which increase emergency myelopoiesis, resulting in the positive feedback of MDSCs (Atretkhany and Drutskaya 2016; Veglia et al. 2021). Finally, after the recruitment, MDSCs can also secrete chemokines such as CXCL1 released by M-MDSCs, which interacts with its receptor CXCR2 present in PMN-MDSCs and thus act in synergy to increase infiltration and promote immunosuppression in the microenvironment (Hangai et al. 2021; Wang et al. 2019).

### Metabolites Related with Immunosuppression

In MDSCs, one of the main immunosuppressive metabolites is Arg1, which converts L-Arginine into L-Ornithine and Urea, entered from the microenvironment through CAT-2B transporters (Groth et al. 2019; Raber et al. 2012). l-Arginine, an amino acid, can be acquired through dietary resources, synthesized endogenously from citrulline during the urea cycle, or obtained via protein turnover (Morris 2007), and is a key amino acid in T cells for the synthesis of the ζ TCR chain. Therefore, the depletion of this amino acid causes a decrease in the TCR function, thus inhibiting T cell proliferation (Raber et al. 2012; Taheri et al. 2001).

Another molecule from this category is the expression of indoleamine-2,3-dioxygenase (IDO) by MDSCs, which degrades tryptophan present in the microenvironment, an essential amino acid involved in the activation of effector T cells and promotes differentiation of regulatory T cells via kynurenine production (Munn and Mellor 2007; Wang et al. 2019; Yang et al. 2020; Zhai et al. 2020).

On the other hand, cysteine is an essential amino acid for glutathione synthesis. There are two ways to obtain cysteine: the entry of exogenous cysteine into the cell through transporters such as alanine-serine-cysteine (ASC) 1 or 2 or via endogenous production through the transsulfuration pathway that transforms methionine into cysteine (Gmünder et al. 1991; Levring et al. 2012). Although these two mechanisms exist, T cells do not produce endogenous cysteine and depend solely on exogenous cysteine-supplied by DC and macrophages (Bannai 1984; Levring et al. 2012). Cysteine/glutamate transporter SLC7A11 is present in DCs and macrophages but not in lymphocytes. SLC7A11 enables the conversion of cystine present in the medium into cysteine. ASC transporter allowing cysteine is released into the media, thereby allowing T cells to use it (Bannai 1984; Gmünder et al. 1991). In contrast to DCs and macrophages, MDSCs also express SLC7A11 but lack ASC transporters. As a result, there is a depletion of cystine from the surrounding medium without the cysteine secretion. The depletion causes lymphocytes to run out of this crucial amino acid, resulting in heightened sensitivity to oxidative stress decreasing their proliferative capacity (Groth et al. 2019; Srivastava et al. 2010).

Finally, MDSCs express ectoenzymes such as CD39 which rapidly convert extracellular ATP to AMP, which is dephosphorylation by CD73 to adenosine (Antonioli et al. 2013; Li et al. 2017). Adenosine has immunosuppressive effects on T lymphocytes. It inhibits the phosphorylation of Zap70, ERK, and Akt in naïve T cells. Adenosine also reduces effector molecules, such as CD95L, CD25, IFN-γ, and TNF-α production, in activated T cells (Li et al. 2017, 2018a; Linnemann et al. 2009).

### Immune Checkpoints Molecules Expressed by MDSCs

MDSC-mediated suppression also encompasses the involvement of immune checkpoint molecules where PD-L1 being a prominent molecule in this context (Ballbach et al. 2017; Iwata et al. 2016), which interacts with the programmed cell death protein 1 (PD-1) present in T lymphocytes, inhibiting proliferation, survival, and effector functions (cytotoxicity, cytokine release), or mediating apoptosis of tumor-specific T cells. Induction of PD-L1 in MDSCs has been correlated with worse disease prognosis in different cancer types (Iwata et al. 2016). Several factors can increase PD-L1 expression in MDSCs, such as HIF1α activation (Wagner Grau 2011), tumor-derived exosomes (Burga et al. 2015; Iwata et al. 2016), and metabolic changes (Prima et al. 2017). MDSCs also express cytotoxic T-lymphocyte antigen (CTLA)-4. Nevertheless, the specific role of PD-L1 in MDSCs remains unclear. However, studies have demonstrated that CTLA-4-blocking antibody reduces MDSC accumulation (Pico de Coaña et al. 2013; Veglia et al. 2021; Yang et al. 2020). Additionally, it is important to mention that the use of natural products combined with immunological checkpoint inhibitors shows significant synergy, as observed in different murine models (Lasso et al. 2020; Zhong et al. 2022). This is a relevant aspect of the use of natural products and is their role as adjuvants to conventional antitumor therapies.

Recently, in the peripheral blood of patients with acute myeloid leukemia, a high expression of VISTA (V-domain Ig-containing suppressor of T cell activation) was observed in MDSCs (Wang et al. 2018), a molecule whose blockade results in decreased suppressive activity in murine models (Le Mercier et al. 2014). On the other hand, the expression of galectin 9 (Sakuishi et al. 2011) and CD155 (Johnston et al. 2014; Harjunpää and Guillerey 2020) has been demonstrated, to provide higher suppressive potential to MDSCs. A Disintegrin and metallopeptidase domain 17 (ADAM17), an enzyme that cleaves the ectodomain of L-selectin, has also been reported to promote a suppressive TME by limiting the recirculation into lymph nodes of CD4^+^ and CD8^+^ T cells, and decreasing CD62 ligand (CD62L) expression (Hanson et al. 2009).

## Current Therapeutic Strategies Against MDSCs

**Fig. 2:**
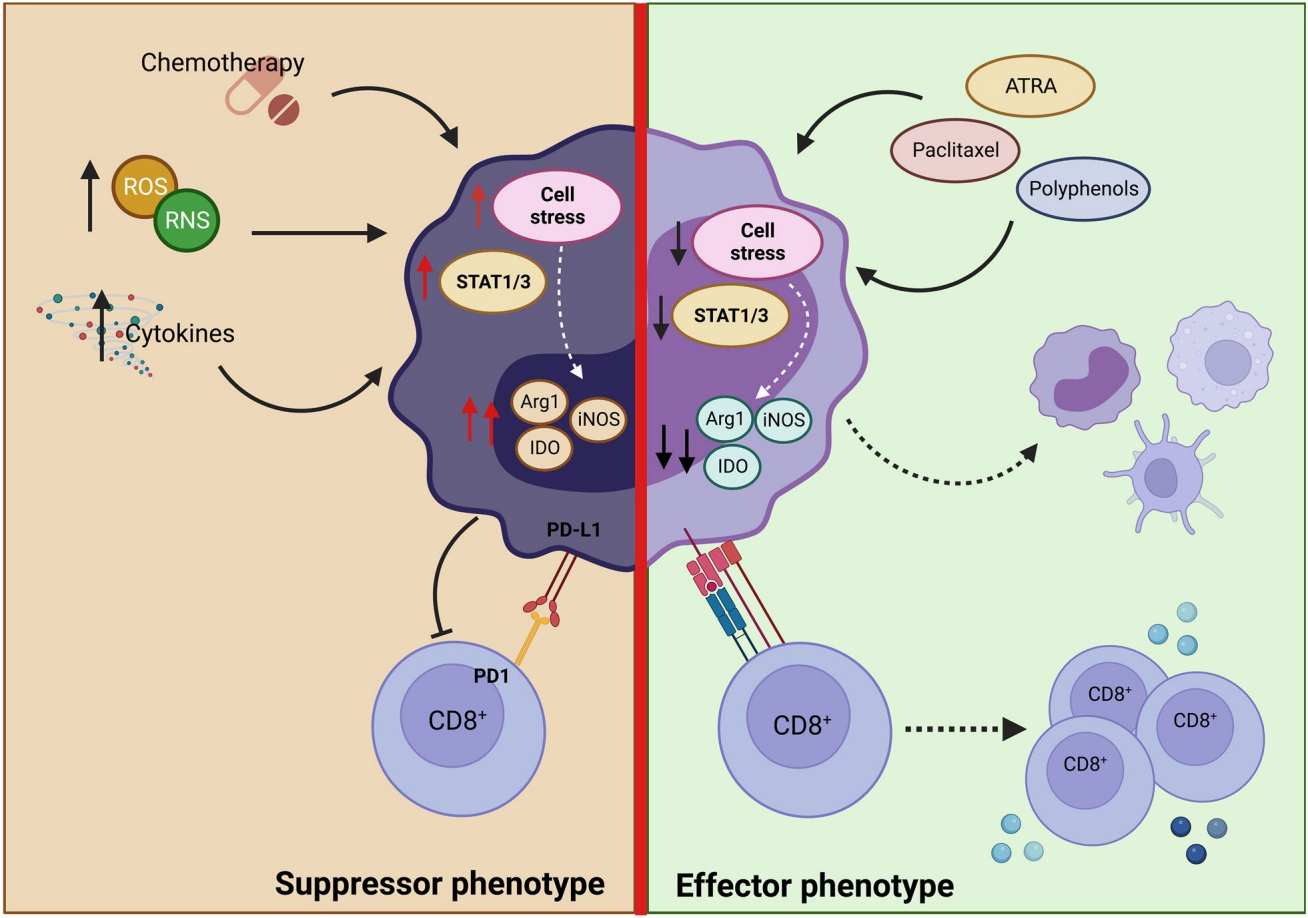
MDSC suppressors functions. MDSCs exposed to conditions, such as a highly oxidative, inflammatory, or post-chemotherapeutic microenvironment, can cause cellular stress, which is associated with an increase in immunosuppressive molecules, such as Arginase 1 (Arg-1), Nitric Oxide Synthase inducible (iNOS), indolamine-2,3-dioxygenase (IDO), and PD-L1. Additionally, this microenvironment also favors the activation of STAT1 and STAT3 in progenitors, increasing the production of these cells in the bone marrow and their subsequent infiltration into the TME, generating a strong suppression of the effector function of the T cells. Despite the above, these pathways can be modulated by different treatments. Among these, we can find ATRA and Paclitaxel, which can favor their differentiation toward other myeloid cells, such as macrophages, monocytes, or dendritic cells. On the other hand, the use of new therapies such as polyphenols due to their chemical composition has an advantage, modulating different cells present in the tumor microenvironment. It has been shown that they decrease STAT3 signaling, causing less MDSC infiltration in the TME. Additionally, due to their important antioxidant activity, they could reduce cellular stress, which would cause attenuation of their suppressive capacity and even acquire the ability to present antigens

MDSCs play a fundamental role in orchestrating immunosuppression within the TME, making them a potential therapeutic target. Currently, the predominant approaches are to deplete MDSCs, inhibit immunosuppressive potential, block infiltration into the TME or take advantage of their high plasticity to convert them into favorable cells for the immune response promoting MDSCs differentiation (Fig. 2) (Aboelella et al. 2021; de Haas et al. 2016; Groth et al. 2019; Law et al. 2020; Mandula and Rodriguez 2021). However, in many approaches, there is not one therapeutic activity directed against a specific target in MDSCs. Instead, they correspond to activities directed at different functions in the organism or in the TME that indirectly impact MDSCs.

It has been observed that small doses of some chemotherapeutic agents can be cytotoxic to MDSCs, in addition to their effect on tumor cells. In this sense, it has been observed that gemcitabine reduces Gr1^+^CD11b^+^ cells in murine models (Suzuki et al. 2005), and additionally, in patients with pancreatic cancer, it generates a transient decrease in PMN-MDSCs (Eriksson et al. 2016). On the other hand, 5-fluorouracil, unlike doxorubicin, oxaliplatin, or cyclophosphamide, reduces spleen- and tumor-infiltrating MDSCs in mice by activating caspases 3 and 7 (Geary et al. 2013; Otsubo et al. 2015; Vincent et al. 2010).

Trying to develop therapies specifically against MDSC, small molecule inhibitors have been developed, such as sunitinib and AG490, to target signaling pathways involved in the MDSCs production. These inhibitors, which act on pathways, such as STAT1 and STAT3, effectively decrease the generation of MDSCs and their suppressive activity (Fig. 1) (Hofer et al. 2021; Ko et al. 2009; Li et al. 2021; Ryan et al. 2020; Wang et al. 2017).

Decitabine, for example, has been shown to decrease MDSCs generation in the BM providing a less immunosuppressive environment (Zhou et al. 2019). On the other hand, it has been seen that treatment with tadalafil, phosphodiesterase inhibitor, in clinical trials decreased MDSCs and increased the specific immune response in patients with metastatic melanoma and head squamous neck cell carcinoma (Califano et al. 2015; Hassel et al. 2017; Weed et al. 2015; Wu et al. 2022). As part of these approaches, peptides that selectively bind to MDSCs have also been developed. These peptides are conjugated with the Fc region of IgG-type antibodies, enabling targeted MDSCs depletion (Qin et al. 2014). On the other hand, some phase I/II clinical studies show that the use of STAT3 inhibitors such as AZD9150 accompanied by anti-PD-L1 antibodies favors a lower accumulation of MDSCs and improves the antitumor response (Hong et al. 2015; Proia et al. 2020; Shastri et al. 2018;). Nevertheless, some chemotherapeutic agents can also produce adverse alterations in the TME related to oxidative stress, ER stress, and cytokine production. This creates a favorable environment for MDSCs expansion (Geary et al. 2013; Mandula and Rodriguez 2021). Taking this into account, one way to counteract this effect is the possibility of combining antitumor drugs or treatments aimed at eliminating cancer cells with therapeutics that affect the recruitment and activation of MDSCs in the TEM.

A second therapeutic approach involves using anti-CXCR2 or CCR2 monoclonal antibodies to reduce MDSCs infiltration in the TME, which have shown some effectiveness in murine models (Draghiciu et al. 2015; Highfill et al. 2014; Zeng et al. 2019). Similar effects were found by inhibition of CXCL12/CXCR4 (Sun et al. 2019). Despite these results, peripheral MDSCs can continue to suppress through mechanisms such as ADAM17. In addition, CCR2 inhibition can affect the antitumor infiltration of macrophages and DCs (Flores-Toro et al. 2020). The use of bevacizumab, anti-VEGF treatment, and inhibitors blocking CSF-1R signaling also decreases MDSCs recruitment (Li et al. 2021).

Some therapies have focused on counteracting the immunosuppressive phenotype of MDSCs. In this sense, iNOS inhibitors such as nitro-L-arginine methyl ester, used with chemotherapy or radiotherapy, improve the reduction of tumor growth in murine models (Granados-Principal et al. 2015; Gschwandtner et al. 2019). It has been seen in clinical trials that the use of inhibitors of Arg1 (INCB001158) and iNOS (ASP9853) in combination with chemotherapy can improve the clinical outcome of the patients (Luke et al. 2016; Pereira et al. 2020). Additionally, immune checkpoint modulators are another useful approach to treating the suppressive activity of MDSCs. Thus, treatment with antibodies against PD-L1 or CTLA-4 generates less accumulation of MDSCs, although the mechanism of action of the latter is unclear (Le Mercier et al. 2014; Pico de Coaña et al. 2013; Veglia et al. 2021; Yang et al. 2020). Antibody blockade of other molecules such as VISTA has been associated with decreased MDSCs and less suppressive activity along with increased DC activation (Grzywa et al. 2020).

Finally, based on the high plasticity of MDSCs, their reprogramming into immuno-stimulatory cells capable of activating the T cell response has been proposed. All-trans-retinoic acid (ATRA), which is a retinoid receptor agonist, has been shown to have the ability to induce MDSCs differentiation into mature myeloid cells, such as DCs and macrophages, in both murine models and patients (Chen et al. 2021; Lee et al. 2012), apparently by ERK 1/2 activation (Kusmartsev et al. 2008; Nefedova et al. 2007). Additionally, in one phase II clinical trial, the combination of ipilimumab plus ATRA showed a significant reduction in circulating MDSCs (Tobin et al. 2018). Similarly, tetra-bromo-cinnamic acid increased Notch signaling by restoring normal myeloid differentiation (Cheng et al. 2014). Paclitaxel is another molecule that induces MDSCs differentiation into DCs through TLR4 signaling (Michels et al. 2012; Sevko et al. 2013). It should be noted that ATRA, in addition to inducing MDSCs maturation, also enhances the redox potential within the cell and modulates ER stress; this last being a key factor in controlling MDSC functionality (Al-Qassab et al. 2018; Molina-Jijón et al. 2015). Table 2 lists these drugs and their activities.

**Table 2:** Therapeutic strategies directed against MDSCs

	Name/product	Mechanism of action	Specie	References
Conventional therapeutics	Gemcitabine	MDSCs depletion	Human Mouse	Eriksson et al. (2016); Suzuki et al. (2005)
	5-Fluoroascil	MDSC depletion by activation of caspases 3 and 7	Mouse	Vincent et al. (2010)
	Sunitinib AZD9150	STAT1 and STAT3 inhibition	Human	Ko et al. (2009); Li et al. (2021); Shastri et al. (2018)
	Decitabine	MDSC depletion	Mouse	Seong et al. (2016); Wagner Grau (2011)
	Tadalafil	MDSC reduction in periphery and tumor	Human	Califano et al. (2015)
	Anti-CXCR2 or Anti-CCR2	Decreased of MDSCs infiltration	Mouse	Sun et al. (2019)
	Bevacizumab	Inhibits VEGF, reduces MDSC infiltration into the tumor	Human	Koinis et al. (2016)
	L-NAME ASP9853 INCB001158	Decrease of immunosuppressive molecules	Human Mouse	Granados-Principal et al. (2015); Kuriakose (2016); Luke et al. (2016)
	Paclitaxel	Differentiation of MDSCs to DCs via TLR-4	Mouse	Michels et al. (2012); Sevko et al. (2013)
	ATRA	MDSCs maturation	Mouse	Al-Qassab et al. (2018); Molina-Jijon et al. (2015)
Natural products	Beta-glucans: Curdlan	MDSC modulation by activation of dectin-1 and NF-κB	Mouse	McIntosh et al. (2005); Tian et al. (2013)
	Fruiting bodies of *Ganoderma lucidum*	MDSCs reduction in spleen and tumor Decrease of immunosuppressive molecules	Mouse	Wang et al. (2020)
	Epigallocatechin-3-gallate (EGCG)	Reduction of intratumoral MDSCs infiltration and differentiation of PMN-MDSCs to mature PMN	Human Mouse	Croce et al. (2010); Santilli et al. (2013); Tachibana (2011)
	Curcumin	Decreased infiltration and expansion of intratumoral MDSCs Decrease of immunosuppressive molecules and IL-6	Mouse	Liu et al 2016; Lu et al. 2016
	Resveratrol	Differentiation of MDSCs to DCs	Mouse	Singh et al. 2007; Zhao et al. 2018
	OLE	ROS/RNS decrease in MDSCs	Mouse	Ashourpour et al. 2016
	P2Et	Decreased of MDSC in spleen and tumor	Mouse	Lasso et al. 2018, 2022

*L-NAME* nitro-L-arginine methyl ester, *OLE* olive leaves extract

## Natural Products as a Novel Therapy Against MDSCs

Despite all the efforts, these therapies have not yet been successful, showing some contradictory results, perhaps associated with the fact that MDSCs are a group of cells with high complexity and heterogeneity, posing a significant barrier to developing molecules that target this population. Consequently, alternative therapies, such as natural products, have been pursued. The development of natural products such as anti-therapeutics has progressed to an increasingly large extent in recent years. This is partly due to some advantages over conventional drugs, such as their lower toxicity and ability to act on multiple molecular targets. However, some of these natural products, particularly those that correspond to plant extracts, may present an incomplete molecular characterization, as well as clear molecular targets on the tumor that have not yet been identified (Atanasov et al. 2021). These and other pros or cons of natural products with conventional pharma products are resumed in Table 3.

**Table 3:** Pros and cons of natural products compared to conventional drugs (Atanasov et al. 2021; Salminen et al. 2018; van Geffen et al. 2022; Wu et al. 2022; Zhang et al. 2022)

	Pros	Cons
Conventional therapeutics	Identification of mechanisms of action can be systematically more robust	Therapy resistance. Conventional treatments over time can select cells resistant to therapy particularly when activities diminishing MDSCs depend on cancer cells
	The previous existence of toxicity studies developed facilitated incorporation of new functions on MDSCs	High toxicity is frequent in conventional drugs
	Low interference with another pharmaceutics	Increase in oxidative stress that in the long term allows an increase in MDSCs
Natural compounds	Diverse chemical structures. Plant complex extracts due to their composition act on multiple targets. Being multi-target is advantageous in the sense of combating the complexity of the heterogeneity of cancer cells and MDSCs present in TME	Lack of standardization. Sometimes targets and molecular mechanisms have not been clearly elucidated
	Its use in traditional medicine can provide information on efficacy and safety	Limited scientific evidence. It is necessary to increase the quantity and quality of the preclinical and clinical evaluation of natural products, particularly in the search for direct activities on the MDSCs
	Reduced toxicity and side effects, partly attributed to their natural origins	Have enough biological material to isolate and characterizing bioactive compounds can be challenging
	Synergism. Natural products have been shown to have synergistic activities when combined with other molecular drugs, including conventional drugs modulating MDSCs generation and/or function	Access to intellectual property rights for bioactivities associated with natural (unmodified) products it is a big challenge
	Higher stiffness of natural products structure molecules can be useful when act on protein–protein interaction involved in MDSCs functions	The generation of structural analogs for exploring structure-biological activity relationships is complex
	Decreased oxidative stress which favors the modulation of MDSCs in the TME	Despite the growing number of investigations involving natural products, very few have clinically evaluated

Within natural products with antitumor activity, there are both complex extracts derived from plants and products of microbial structures, such as polysaccharides. Traditionally, polysaccharides have been attributed to the ability to generate a response mediated by pattern recognition receptors, such as TLR4, CD14, Detectin-1, or mannose receptors (Li et al. 2018b), which normally cause non-specific activation of the immune response.

Among the polysaccharides of microorganisms, we can find beta-glucans, which are components of the surface of bacteria and fungi (Tian et al. 2013). According to recent research, beta-glucans modulate the immune response and are cytotoxic on tumor cells (Harnack et al. 2009; Kim et al. 2011). Additionally, it has been demonstrated using in vitro assays that these glucans can induce MDSCs maturation through the dectin-1 and NF-κB pathways, decreasing their suppressive activity and improving the response of CD4^+^ and CD8^+^ T cells (Tian et al. 2013). On the other hand, Curdlan, a compound of linear b-(1,3)-glucosidic bonds from *Alcaligenes faecalis* (McIntosh et al. 2005), has a direct effect on MDSCs, reducing its suppressive activity, reflected in a decreased expression of Arg1, a reduced ability to suppress T lymphocytes and a lower MDSCs infiltration in the TME (Rui et al. 2016). Similarly, it has been shown that polysaccharides derived from the fruiting bodies of *Ganoderma lucidum* one polypore fungi showed an antitumor effect that was accompanied by a lower infiltration of MDSCs in the spleen and in TME (Wang et al. 2020). Additionally, MDSCs showed IDO, iNOS, and Arg1 decreasing, all mediated by the CARD9-NF-κB-IDO pathway (Wang et al. 2020). All these activities reflect modulation mechanisms of the innate immune response through primary metabolites of microorganisms/fungi and could be equally extended to molecules derived from superior organisms such as plants.

On the other hand, several natural compounds are secondary metabolites derived from plants. They have diverse chemical structures and can impact various molecular targets, exhibiting a wide range of biological activities (Newman and Cragg 2016). These compounds include alkaloids, terpenes, glucosinolates, and polyphenols, which play a prominent role in cancer treatment (Gomez-Cadena et al. 2020; Nwokeji et al. 2016). Due to their chemical composition, natural products exhibit distinct behaviors depending on the microenvironment. This characteristic holds particular significance in cancer therapy as it allows them to modulate TME and attack the tumor without affecting normal cells, providing them with an advantage over conventional chemotherapeutic agents. For example, polyphenols neutralize free radicals by donating an electron or a hydrogen atom (Loftus et al. 2018), and although ROS are not per se a specific target in tumor cells, the fact that these cells present a higher level of ROS generates a differential response in malignant cells compared to their normal counterparts when treated with antioxidants. The latter is important but it must be considered that the use of antioxidant compounds in cancer therapy presents a duality since it has been seen that they can reduce immunosuppression within the TME; however, some studies have shown that they can increase metastasis in some patients (Cockfield and Schafer 2019). In recent years, polyphenols have been shown to modulate oxidative stress in the TME. This represents a substantial therapeutic advantage since a high level of ROS generates favorable conditions to inhibit the immune response and increase MDSCs activation (Mandula and Rodriguez 2021). Additionally, antioxidants such as NAC decrease ER stress in the TME (Cao et al. 2019; Thevenot et al. 2014). Therefore, antioxidants are the key in the search for therapeutic alternatives that may influence MDSC-mediated suppression (Fig. 2).

There are numerous examples highlighting the activity of polyphenols on MDSCs. One of the most common compounds is the epigallocatechin-3-gallate (EGCG) in green tea, which has antioxidant and anti-inflammatory properties (Tachibana 2011). Polyphenol E, synthesized from EGCG, was shown to have an in vitro effect on neuroblastoma cells and a protective effect in mouse models (Tachibana 2011; Santilli et al. 2013). On the other hand, it has been observed that Polyphenol E affects the chemotaxis of MDSCs, resulting in less infiltration, and differentiation into cells like mature PMNs. This change was mediated by downstream signaling of the laminin and G-CSF receptor (Condamine and Gabrilovich 2011; Croce et al. 2010; Santilli et al. 2013; Tachibana 2011). Similarly, Curcumin a highly pleiotropic molecule derived from turmeric has been shown to inhibit growth and generate apoptosis of different tumor lines (Liu et al. 2016), and modulate signaling pathways, such as JAT-STAT, NF-κB (Vallianou et al. 2015), MAPK (Zhao et al. 2015), and VEGF (Fu et al. 2015). Curcumin treatment reduces MDSC accumulation in melanoma (Lu et al. 2016). On the other hand, in lung cancer, decreased MDSC infiltration was observed accompanied by decreased immunosuppressive molecules and downregulation of IL-6, which affects MDSCs expansion and activation (Liu et al. 2016). In addition to the molecules mentioned above, resveratrol also acts in different tumor cell lines capable of activating macrophages and effector T cells (Buttari et al. 2014; Singh et al. 2007). Furthermore, resveratrol promotes the M-MDSCs differentiation into CD11c^+^ and F4/80^+^ cells and decreases the proportion of PMN-MDSCs (Zhao et al. 2018).

Many studies of activities related to natural products on MDSC result from indirect activities on TME that favor the decrease of the infiltration and activation of MDSC in the tumor. However, there are some works where a direct action on MDSCs is shown, as is the case of Icariin, a compound obtained from Herba Epimedii of traditional Chinese medicine, where they show that direct exposure of MDSCs to this compound generated their differentiation in DCs and macrophages. These results were accompanied by a decrease in MDSC in the spleen of mice with 4T1 breast cancer tumors treated with Icariin (Zhou et al. 2011). The effects of these natural products on MDSCs may also be the consequence of clearly elucidated activities on cancer cells. In this sense, berberine, a natural component of traditional Chinese medicine (an isoquinoline quaternary alkaloid derived from *Coptis chinensis*), is capable of promoting the proteasome-dependent degradation of PD-L1 on tumor cells and thus increasing the intra-tumoral infiltrate of T cells accompanied by a decrease in MDSCs in a Lewis tumor xenograft mice (Liu et al. 2020). These are some examples to show activities of natural compounds directed specifically to affect MDSCs.

Other natural products with high antitumor potential are complex extracts obtained from plants. They have several compounds, which can act on multiple targets in cancer cells or into the TME, showing a broad spectrum of biological activities to control cancer (Hopkins 2008). In this regard, the extract containing poly-acetylene glycosides from the medicinal plant *Bidens pilosa* has been shown to suppress metastasis and intra-tumoral PMN-MDSC accumulation and functionality in the 4T1 mouse model of breast cancer. Within the chemical characterization of this extract, three main types of compounds were found (2-β-d-glucopyranosyloxy-1-hydroxy-5(E)-tridecene-7,9,11-triyne, 2-d-glucopyranosyloxy-1-hydroxytrideca-5,7,9,11-tetrayne, and 3-β-d-glucopyranosyloxy-1-hydroxy-6(E)-tetradecene-8,10,12-triyne), which showed excellent bioavailability when administered orally (Wei et al. 2016). On the other hand, our group has progressed in the characterization of a complex extract called P2Et, from the *Caesalpinia spinosa* plant, which is enriched in polyphenols, such as methyl gallate, gallic acid, and ethyl gallate (Sandoval et al. 2016)*.* This extract has been shown to decrease MDSC infiltration in the spleen and tumor in murine melanoma and breast cancer in vivo models (Lasso et al. 2018, 2022). The mechanisms by which this occurs are still unclear. However, other different studies have shown that treatment with polyphenols such as curcumin–polyethylene glycol (CUR-PEG) showed the modulation of STAT3 in MDSCs, which was associated with a decrease in its infiltration in the spleen and tumor (Lu et al. 2016). As describe above, an exciting aspect of the activities on the MDSC compartment of the natural extracts is the modulation of the oxidative stress they can exert on the tumor’s cancer cells. In this sense, we recently showed that the P2Et extract, unlike an extract from the *Tillandsia usneoides* plant, decreases tumor growth using the B16-F10 murine melanoma model. This effect on tumor growth was accompanied by decreased of PMN-MDSCs from draining lymph nodes and tumor in mice treated with P2Et, but not with *T. usneoides* extract. It was shown using in vitro assays that P2Et extract, unlike that of *T. usneoides*, decreased ROS in B16-F10 tumor cells (Lasso et al. 2022). The P2Et extract, being an extract rich in polyphenols, such as gallic acid and ethyl gallate, has shown significant antioxidant activity in different tumor lines that could be associated with the in vivo activity (Ballesteros-Ramírez et al. 2020; Lasso et al. 2018). Additionally, P2Et has shown significant antitumor activity in both in vitro and in vivo experiments (Gómez Cadena et al. 2013; Lasso et al. 2018; Urueña et al. 2013). Something that stands out about P2Et is its ability to modulate autophagy and reticulum stress in tumor cells (Gomez-Cadena et al. 2015; Prieto et al. 2019), causing its apoptosis and release of damage-associated molecular patterns associated with immunogenic cell death acting by calcium-dependent mechanisms without increasing oxidative stress (Prieto et al. 2019). Taken together, these data reflect that P2Et could have a promising impact on the modulation of MDSCs. Although complex plant extracts represent a great challenge given their complexity in terms of their molecular characterization, given their poly-molecular nature, they are a very promising alternative to be used in complex diseases such as cancer, being able to act on different molecular targets, affecting different mechanisms on cancer. However, great efforts must be made to characterize in a deeper way the mechanisms of action of these extracts.

One of the difficulties in developing therapies aimed at reducing the generation, accumulation, and activation of MDSCs in the TME comes from the need for clinical trials to accurately assess these activities. Much of the available data come from clinical trials where the observation of an effect on MDSCs occurs by accident when analyzing the immune cell compartment of patients (Tobin et al. 2017). Considering the effectiveness of accidental MDSC depletion, currently more than five Phase 1b/2 clinical trials are in progress to target MDSCs (Law et al. 2020). This fact is magnified for clinical trials involving natural products, particularly those corresponding to complex mixtures used as phyto-therapeutics. MDSCs are susceptible to modification by ATRA, in aggressive melanoma. For example, the use of ATRA in combination with pembrolizumab showed a favorable tolerability and a high response rate on patients, with lower frequency of circulating MDSCs showing that targeting MDSCs using natural drugs is an attractive mechanism to enhance the efficacy of immunotherapies (Tobin et al. 2023).

## Perspectives and Conclusions

The complexity and the heterogeneity of MDSCs constitute a barrier to the development of specific molecules that act on this cell population. Although some chemotherapeutics and synthetic molecules have been developed and progressed to clinical studies, a viable and effective alternative that allows attacking these cells without collateral damage has not yet been observed. For example, during the use of chemotherapeutics, although some modulate or deplete them, in the long term, they induce a more adverse TME, which ends up generating greater stress and a favorable niche for the generation of these MDSCs. In the long term, this can be translated as a therapeutic failure, considering that MDSCs are important in allowing the immunological escape of tumor cells. For this reason, the need for a treatment that goes beyond simply killing these cells is created, an alternative is needed that modulates the TME in such a way that it does not favor the production and activation of MDSCs, but rather takes advantage of their plasticity transforming them to a less-suppressive phenotype that is even capable of increasing the immune response.

## Data Availability

Not applicable in this case.
